# Sex as a biological variable in nonclinical studies: Bridging scientific rigor, animal welfare, and regulatory expectations

**DOI:** 10.1002/ame2.70182

**Published:** 2026-03-26

**Authors:** Kenta Onuma, Masaki Watanabe, Nobuya Sasaki

**Affiliations:** ^1^ Laboratory of Laboratory Animal Science and Medicine, School of Veterinary Medicine Kitasato University Towada Japan

**Keywords:** animal welfare, nonclinical studies, pharmacokinetics, regulatory science, sex as a biological variable

## Abstract

Nonclinical studies in animal models have often treated male animals as the default, based on the assumption that estrous‐cycle hormonal variation in females increases variability and complicates interpretation. This convention is a structural limitation in study design, particularly in therapeutic areas where sex differences in pharmacokinetics (PK), pharmacodynamics (PD), and toxicity are substantial and clinically relevant. Clinical experience shows higher adverse drug reaction rates in women and recurring examples of sex‐biased toxicities, underscoring the translational cost of limited sex consideration early in development. Sex differences in nonclinical PK and toxicity arise from defined mechanisms, including sex‐dependent growth hormone secretion patterns that shape hepatic and renal enzyme/transporter expression, as well as sex‐specific determinants of gastrointestinal absorption and cardiac electrophysiology. Omics analyses further show that sex effects are embedded in system‐wide molecular networks across immune, metabolic, cardiovascular, renal, and neural systems, supporting a shift from descriptive to predictive and stratified animal study designs. Sex bias is also an animal welfare concern: male‐only strategies can generate surpluses of unused females in breeding colonies, challenging the 3Rs—particularly Reduction—when evaluated at the project or facility level. Regulatory expectations are converging through International Council for Harmonization (ICH) principles, the National Institutes of Health (NIH) sex‐as‐a‐biological‐variable (SABV) policy, and reporting frameworks such as Sex and Gender Equity in Research (SAGER), with increasing emphasis on transparent sex reporting and analysis across nonclinical evidence streams. This review integrates scientific, welfare, and regulatory perspectives to provide a practical framework for sex‐aware nonclinical study design, enhancing translational validity and ethical rigor.

## Graphical abstract

Sex as a biological variable (SABV) is a critical determinant of pharmacokinetics, pharmacodynamics, and toxicity in nonclinical studies. This review highlights how inappropriate neglect of sex differences compromises animal welfare, scientific reproducibility, and regulatory compliance. We propose practical strategies to integrate SABV into nonclinical study design while maintaining efficiency and reduction in animal use.

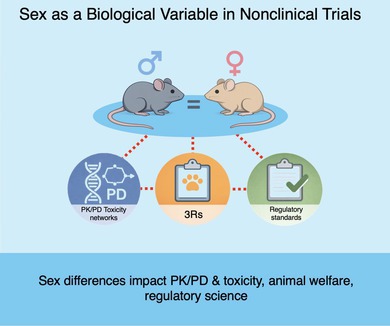

## 1 INTRODUCTION

In drug development, nonclinical studies have long used male experimental animals as the de facto standard. Particularly during basic research and exploratory toxicity testing, female animals are often excluded due to concerns that hormonal fluctuations associated with the estrous cycle increase data variability and complicate analysis.^1^ Although this approach may simplify experiments and reduce short‐term costs, it is increasingly clear that it compromises the reproducibility and clinical translatability of nonclinical data.

This concern is especially relevant in therapeutic areas such as central nervous system disorders and immunomodulation, where sex differences in drug response are well documented. Clinically, adverse drug reactions (ADRs) are reported more frequently in women than in men, with incidence rates estimated to be 1.5–1.7 times higher.^2^ Moreover, a substantial proportion of drugs withdrawn from the market have caused more severe adverse events in women.^3^ These observations reflect a structural gap. Sex differences are often not systematically evaluated as biological variables during early nonclinical and clinical development. Sex differences are not merely experimental noise; they can directly influence pharmacokinetics (PK), pharmacodynamics (PD), and toxicity mechanisms. Therefore, they should be incorporated into nonclinical study design as core determinants of interpretability and translatability.

From the perspective of laboratory animal veterinarians and facility managers, this issue is also closely linked to animal welfare. Male‐only study designs may appear to reduce animal use per experiment. However, they can structurally generate a surplus of female animals in breeding colonies, which are often not used in research and are ultimately culled.^4^ This creates a practical dilemma between adhering to the 3Rs (particularly Reduction) and maintaining research practices that ignore sex as a biological variable. Scientifically unjustified sex selection has become difficult to justify under contemporary welfare standards and is discouraged by international frameworks such as Directive 2010/63/EU, which emphasizes appropriate and balanced animal use.

Globally, the integration of sex as a biological variable (SABV) has become an irreversible trend. Since 2016, the US National Institutes of Health (NIH) has required SABV consideration in vertebrate animal research as a condition for funding.^5^, ^6^ In scientific reporting, the Sex and Gender Equity in Research (SAGER) guidelines recommend explicit reporting and sex‐stratified analyses in study design, analysis, and manuscripts.^7^ In this review, “sex” refers to biological attributes (e.g., chromosomal complement, endocrine status, anatomy), whereas “gender” refers to sociocultural constructs relevant primarily to clinical and epidemiological contexts. Accordingly, this review focuses on biological sex differences in animal‐based nonclinical studies.

Most prior reviews emphasize either mechanistic physiology/molecular biology or clinical/epidemiological evidence. In contrast, reviews that integrate scientific rigor, animal welfare, and regulatory expectations at the operational level of nonclinical toxicology—addressing study design, animal use, endpoints, and regulatory interaction—remain limited.

Therefore, this review aims to: (i) systematically summarize the current status of sex consideration in nonclinical drug development across PK, toxicity evaluation, welfare, and regulatory trends; (ii) address common practical barriers to including female animals (e.g., cost, operational burden) using scientific, ethical, and regulatory arguments; and (iii) provide implementable approaches for pharmaceutical developers, laboratory animal professionals, toxicologists, and regulatory‐science stakeholders. Unlike previous SABV reviews that focus primarily on physiology or clinical epidemiology, this review uniquely integrates mechanistic biology, animal welfare logistics, and regulatory implementation into an operational framework directly applicable to nonclinical toxicology practice.

## 2 SCIENTIFIC BASIS OF SEX DIFFERENCES

### 2.1 Sex Differences in Pharmacokinetics and Toxicology

**FIGURE 1:**
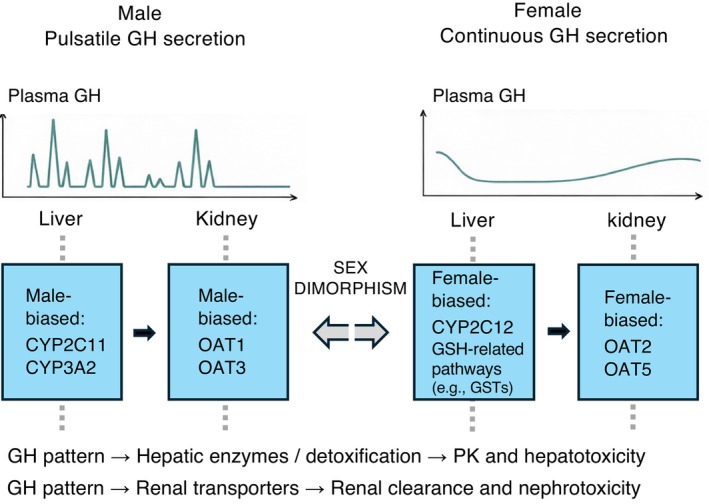
Representative molecular determinants of sex differences in pharmacokinetics and toxicity in laboratory rodents (rat‐centered). This schematic summarizes a growth hormone (GH)–pattern–associated framework for sex differences observed in nonclinical models, with representative examples drawn primarily from rat studies. (Left) In males, pulsatile GH secretion is associated with higher expression of selected hepatic drug‐metabolizing enzymes (e.g., CYP2C11, CYP3A2) and renal transporters (e.g., OAT1, OAT3), which may contribute to sex‐dependent differences in drug metabolism and renal clearance. (Right) In females, more continuous GH secretion may be associated with higher CYP2C12 and phase II detoxification programs (including GSH‐related pathways), which in some contexts is consistent with relatively greater detoxification capacity, and with higher expression of selected renal transporters (e.g., OAT2, OAT5). Dotted lines indicate conceptual mapping (representative associations) rather than direct causality. Isoforms and the magnitude of sex differences vary by species and experimental conditions.

Representative determinants relevant to this section are summarized in Table 1 and illustrated in Figure 1.

**TABLE 1:** Representative molecular determinants of sex differences in pharmacokinetics and toxicity in laboratory rodents (rat‐centered).

Category	Molecule/factor	Direction of sex difference	Primary species	Effect/representative drug examples	References
Endocrine regulation	GH secretion pattern	Male: pulsatile; Female: more continuous	Multiple (rodents‐centered)	PK/Tox: upstream regulator of hepatic/renal enzyme and transporter expression; bias in exposure and organ‐specific toxicity interpretation	8, 9, 10
Hepatic metabolic enzymes	CYP2C11	Male > Female	Rat	PK: male‐predominant metabolism; lower exposure	^11^
Hepatic metabolic enzymes	CYP3A2	Male > Female	Rat	PK: multi‐drug metabolism; PK differences	^11^
Hepatic metabolic enzymes	CYP2C12	Female > Male	Rat	PK: female‐predominant metabolism	^11^
Hepatic metabolic enzymes	CYP2A1	Female > Male	Rat	PK: female‐predominant metabolism	^11^
Renal transporters	OAT1/OAT3	Male > Female	Rat	Tox: cisplatin nephrotoxicity; higher male susceptibility (higher OAT1/3)	^12^
Renal transporters	OAT2	Female > Male (rat; species/strain‐dependent)	Rat	PK/tox: organic anion handling; may influence renal clearance of selected anions (substrate‐dependent)	^13^
Renal transporters	OAT5	Female > Male (rodents; androgen‐suppressed)	Rat/Mouse	PK/tox: organic anion exchange; reported substrates include steroid sulfates; may modulate OTA handling	^14^
Detoxification capacity	Glutathione (GSH)	Female > Male	Mouse	Tox: acetaminophen hepatotoxicity; higher female resistance (higher GSH capacity)	^15^
Gastrointestinal motility	Whole gut transit time	Male < Female (condition‐dependent)	Mouse	PK: absorption rate/Cmax differences; interacts with fasting, time‐of‐day, strain	16, 17
Absorption barrier	P‐gp (MDR1)	Male > Female (strain‐dependent)	Rat	PK: sex differences in oral absorption (P‐gp expression varies by strain)	^18^
Cardiotoxicity related	Ventricular APD	Female > Male	Rabbit	Tox: QT prolongation; higher female arrhythmia susceptibility	19, 20
Cardiotoxicity related	Ventricular APD/current density	Female > Male	Dog	Tox: QTc sex difference may be limited; sex‐dependent electrophysiological parameters (e.g., current density, APD)	^21^
Hepatic metabolic enzymes	Cyp2b/Cyp3a isoforms	Female > Male	Mouse	PK: female‐predominant hepatic expression; affects xenobiotic metabolic rate	^8^
Higher species/translation	P450 activity (selected isoforms)	Context‐dependent	Cynomolgus monkey	PK: sex‐dependent differences in physiology and some CYP activities; translation relevance	^22^

*Note*: This table summarizes key determinants of sex differences in pharmacokinetics (PK) and toxicity in laboratory rodents, mainly rats. Sex‐biased expression of hepatic drug‐metabolizing enzymes (e.g., CYP2C11, CYP2C12, CYP3A2, CYP2A1) and renal transporters (e.g., OAT1, OAT3) is largely shaped by sexually dimorphic growth hormone (GH) secretion patterns, which may drive differences in drug metabolism, exposure, and organ‐specific toxicity. Examples include male‐predominant cisplatin nephrotoxicity (higher OAT1/OAT3) and female‐biased detoxification capacity (enhanced hepatic glutathione pathways). Isoforms and effect sizes may vary across species, including humans.

Abbreviations: CYP, cytochrome P450; GH, growth hormone; OAT, organic anion transporter; PD, pharmacodynamics; PK, pharmacokinetics.

A major driver of sex differences in PK and toxicity in experimental animals is sex‐dependent growth hormone (GH) secretion patterns, which regulate enzyme/transporter expression and physiological traits in multiple organs, including the liver and kidneys.^8^, ^9^, ^10^ In many species, pulsatile GH secretion in males versus more continuous secretion in females shapes hepatic and renal transcriptional programs relevant to drug metabolism and transport.

In the liver, cytochrome P450 (CYP) expression shows pronounced sex differences. For example, in rats, CYP2C11 and CYP3A2 are typically male‐predominant, whereas CYP2C12 and CYP2A1 are female‐predominant.^11^ In the kidney, higher expression of organic anion transporters (OAT1/OAT3) in males can contribute to male‐predominant nephrotoxicity for certain compounds, such as cisplatin.^12^

Collectively, these GH‐pattern–associated differences are consistent with sex‐dependent variation in drug metabolism and renal clearance in rodents; however, the direction and magnitude of sex effects vary by species/strain and experimental conditions. In females, selected renal transporters (e.g., OAT2, OAT5) may also show higher expression than in males in some rodent species/strains.^13^, ^14^ In mice, female‐biased phase II detoxification programs (including GSH‐related pathways) have been reported in specific contexts (e.g., acetaminophen models).^15^ Sex differences are also observed in gastrointestinal motility and drug absorption barriers like P‐glycoprotein (P‐gp/MDR1). These can interact with strain effects, circadian rhythms, and fasting conditions.^16^, ^17^, ^18^ In cardiotoxicity assessment, rabbits exhibit sex differences that resemble humans (e.g., prolonged action potential duration in females), whereas dogs may show limited sex differences in QTc but still exhibit sex‐dependent variation in electrophysiological parameters such as current density and action potential duration.^19^, ^20^, ^21^ Overall, these data indicate that PK/toxicity studies ignoring sex can yield biased exposure assessments and toxicity interpretations.

In mice, significant sex differences occur in immune responses and liver metabolism, such as the female‐predominant expression of certain Cyp2b and Cyp3a isoforms, which influences the metabolic rate of numerous xenobiotics.^8^ These findings also extend to non‐human primates; for instance, cynomolgus monkeys show sex‐dependent differences in renal physiology and certain cytochrome P450 activities.^22^ This suggests that even in higher species, sex remains a core variable for clinical translation. Therefore, robust nonclinical evaluation across multiple species requires a systematic approach to SABV to ensure the safety and efficacy of potential therapeutics.

### 2.2 Omics‐based mechanisms of sex differences

The understanding of sex differences has evolved from hormone‐centric explanations to recognizing system‐wide molecular network architectures. This shift has been accelerated by omics technologies—transcriptomics, epigenomics, proteomics, and metabolomics—which enable quantitative visualization of organ‐spanning and cell‐type‐specific sex differences. Here, we organize integrative evidence, with omics as a core component, into two layers: (i) system‐wide conserved principles and (ii) evidence directly relevant to drug development, corresponding to Tables 2 and 3, respectively.

**TABLE 2:** Conserved system‐wide molecular networks underlying sex differences supported by integrative evidence (including omics analyses).

Category/biological system	Primary focus	Subject	Title	Key findings/overview	References
Autoimmunity; hormones; sex chromosomes	Epidemiology + mechanism	Human + Animal model	Gender differences in autoimmune disease	Summarizes sex and gender differences across major autoimmune diseases, emphasizing roles of hormones, sex chromosomes, and immune regulation	^23^
Cardiology (general)	Epidemiology + mechanism + treatment	Human	Sex and gender differences in cardiovascular disease	Synthesizes evidence that sex and gender modify cardiovascular risk factors, disease phenotypes, and responses to treatment	^24^
Cardiology/heart failure	Epidemiology + mechanism + treatment	Human	Impact of sex and gender on heart failure	Reviews sex and gender effects in heart failure epidemiology, pathophysiology, and responses to evidence‐based therapies; highlights gaps in current guidelines	^25^
Endocrinology/energy metabolism	Mechanism	Human + Animal model	Sex differences in energy metabolism: natural selection, mechanisms and consequences	Connects evolutionary theory to molecular mechanisms explaining pervasive sex differences in energy metabolism across tissues	^26^
Endocrinology/metabolism	Mechanism	Human	Sex differences in skeletal muscle metabolism in exercise and type 2 diabetes mellitus	Reviews sex‐specific regulation of skeletal muscle metabolism in exercise and type 2 diabetes and discusses implications for therapy	^27^
Hepatology/metabolic disease	Epidemiology + mechanism	Human + Animal model	Gender differences in nonalcoholic fatty liver disease	Explains how sex hormones, metabolism, and comorbidities contribute to sex differences in NAFLD prevalence, progression, and outcomes	^28^
Immunity; vaccines; hormones; microbiome	Epidemiology + mechanism	Human + Animal model	Sex differences in immune responses	Reviews sex differences in innate and adaptive immunity, including responses to infection, vaccination, and autoimmunity	^29^
Immunology/infectious disease	Epidemiology + mechanism	Human + Animal model	Considering how biological sex impacts immune responses in COVID‐19	Discusses how biological sex shapes immune responses and clinical outcomes in COVID‐19, with lessons for future infectious disease threats	^30^
Immunology; transcriptomics; RNA‐seq; ATAC‐seq	Mechanism	Animal model	ImmGen report: sexual dimorphism in the immune system transcriptome	Uses ImmGen RNA‐seq and chromatin‐accessibility resources to map sexual dimorphism across immune cell types as a systems‐level reference	^31^
Microbiome/cardiometabolic	Epidemiology + mechanism	Human + Animal model	Sex differences in the association between gut microbiome and hypertension	Highlights sex‐specific links between gut microbiota composition, blood pressure regulation, and hypertension risk	^32^
Microbiome; endocrinology; metabolism	Mechanism	Human + Animal model	How biological sex of the host shapes its gut microbiota	Reviews how biological sex shapes the gut microbiota and how these differences may influence immunity, metabolism, and disease risk	^33^
Nephrology	Epidemiology + mechanism	Human + Animal model	Sex differences in kidney health and disease	Overviews sex differences in kidney structure and function and their relevance across acute kidney injury to chronic kidney disease	^34^
Nephrology	Epidemiology	Human	Sex and gender disparities in the epidemiology and outcomes of chronic kidney disease	Synthesizes global epidemiologic evidence on sex and gender differences in CKD incidence, progression, and mortality	^35^
Nephrology/methods	Mechanism + methods	Human + Animal model	Embracing sex‐specific differences in engineered kidney models	Argues engineered kidney models should incorporate sex‐specific parameters to better capture physiology, disease mechanisms, and drug responses	^36^
Neurology (Alzheimer's)	Mechanism	Human + Animal model	Mechanisms of sex differences in Alzheimer's disease	Synthesizes clinical and experimental evidence for women's higher Alzheimer's disease risk, highlighting hormonal, genetic, and metabolic mechanisms	^37^
Neurology/brain	Mechanism	Human	Single‐cell analysis of sex/gender differences in the brain	Uses single‐cell approaches to map sex and gender differences across the human brain during development and aging, highlighting cell type–specific signatures	^38^
Neurology; sex differences; scoping review	Epidemiology + mechanism	Human	Sex differences in neurology: a scoping review	Scoping review mapping sex differences across neurological disorders and organizing findings into themes and priorities for future research	^39^
Policy/methods	Methods	Animal model	An endocrine society scientific statement: considering sex as a biological variable in preclinical research	Endocrine Society scientific statement providing practical guidance on considering sex as a biological variable in preclinical research	^40^

*Note*: This table summarizes representative studies supporting the concept that sex differences are embedded in conserved molecular network architectures across multiple biological systems. Major domains include immune/autoimmune regulation, cardiovascular physiology, metabolic/endocrine systems, neurobiology, and renal function. Studies integrate evidence from bulk and single‐cell omics and large‐scale cohort analyses, highlighting sex‐dependent gene expression, signaling pathways, and regulatory networks, and supporting the view that sex differences are intrinsic biological properties rather than isolated hormonal effects.

**TABLE 3:** Evidence of sex differences with direct implications for drug development and toxicity assessment.

Category	Primary focus	Species/model	Title	Overview	References
Cardiology/hypertension	Mechanism (hypertension)	Human only	Sex differences in mechanisms of hypertension in humans	Reviews human data on sex‐specific mechanisms of hypertension, including vascular, renal and neurohormonal pathways	^41^
Cardiology/hypertension	Primary care: treatment + epidemiology	Human only	Hypertension: sex‐related differences in drug treatment, prevalence and blood pressure control in primary care	Describes sex‐related differences in hypertension prevalence, prescribing patterns and blood pressure control in primary care settings	^42^
Nephrology/transporters	Mechanism (renal transporters)	Multi‐species	Sex differences in renal transporters	Summarizes sex differences in renal drug and solute transporters and their implications for pharmacokinetics, efficacy and toxicity	^43^
Neuroscience/pain	Mechanism + methods (pain research bias)	Multi‐species	Qualitative sex differences in pain processing: emerging evidence of a biased literature	Critically examines how experimental design and reporting biases have shaped current views on qualitative sex differences in pain processing	^44^
Onco‐immunology	Treatment (immunotherapy) + mechanism	Multi‐species	Advances in sex disparities for cancer immunotherapy	Outlines sex disparities in responses and adverse events with cancer immunotherapies and explores underlying immune and hormonal mechanisms	^45^
Oncology/clinical trials	Treatment outcomes + epidemiology	Human only	Outcome differences by sex in oncology clinical trials	Analyzes oncology clinical trials to identify sex‐based differences in efficacy and toxicity, revealing under‐recognized treatment gaps	^46^
Oncology/toxicity	Treatment (toxicity) + mechanism	Multi‐species	Removing barriers to address sex differences in anticancer drug toxicity	Discusses biological and structural reasons why women frequently experience greater anticancer drug toxicity and proposes strategies to reduce this gap	^47^
Neuroscience/pain	Mechanism (pain pathways)	Multi‐species	Sex differences in pain along the neuraxis	Integrates preclinical and clinical evidence for sex differences in pain processing along the neuraxis, from peripheral nociceptors to the brain	^48^
Pharmacology	Mechanism (PK/PD review)	Multi‐species	Sex differences in pharmacokinetics and pharmacodynamics	Provides a classic overview of sex differences in pharmacokinetics and pharmacodynamics across drug classes in humans and animals	^49^
Pharmacology/PK‐PD	Epidemiology + mechanism (PK/ADR)	Human only	Sex differences in pharmacokinetics predict adverse drug reactions in women	Shows that higher and more prolonged drug exposure in women often predicts their greater risk of adverse drug reactions, arguing for sex‐aware dosing	^50^

*Note*: This table compiles key clinical and translational studies demonstrating sex differences relevant to drug development, safety evaluation, and regulatory science. Examples include sex‐biased drug exposure, adverse drug reaction frequency, and sex‐dependent efficacy/toxicity patterns in areas such as cardiovascular/hypertension, oncology/immunotherapy, pain research, and renal transporter biology. Where available, links to pharmacokinetic, immunological, or molecular mechanisms are noted, illustrating how systems‐level sex differences translate into clinically meaningful variability in drug response and safety outcomes.

Abbreviations: ADR, adverse drug reaction; PD, pharmacodynamics, PK, pharmacokinetics.

#### 2.2.1 Conserved system‐wide sex‐specific networks

Single‐cell RNA sequencing and epigenomic profiling indicate that sex differences represent broadly conserved network properties rather than organ‐limited phenomena.

In the nervous system, sex‐associated gene expression signatures have been detected across diverse cell lineages from development through aging, including neuronal subtypes, glia, and immune‐related cells. Notably, although the identity of sex‐biased genes varies with age and disease context, their functional enrichment patterns remain highly consistent, indicating convergence at the pathway and network level rather than at the single‐gene level.^38^ Integrative analyses in neurodegeneration have linked female‐biased risk to the combined influence of endocrine transitions, genetic factors, and sex‐dependent lipid/energy metabolism networks.^37^ Consequently, in neuropharmacology, nonclinical studies should consider sex‐stratified assessment of network‐relevant endpoints, such as neuroinflammatory cytokine levels (e.g., interleukin‐1β [IL‐1β], tumor necrosis factor‐alpha [TNF‐α]) or markers of oxidative stress in target brain regions, to better predict differential therapeutic responses or neurotoxicity risks.^37^

In immune systems, large‐scale transcriptomic and chromatin accessibility datasets demonstrate consistent sex differences across immune cell subsets, involving X‐linked gene regulation, cytokine signaling, and interferon responses.^29^ These network differences offer a mechanistic context for the female predominance in autoimmune diseases and sex‐dependent vaccine and infection responses.^23^ In infectious diseases, sex‐biased inflammatory trajectories have been associated with clinical outcomes in COVID‐19. They provide a prominent example where immune‐network sex differences translate into differential morbidity.^30^ Therefore, for immunomodulatory drugs, evaluating sex‐specific immune endpoints—such as interferon‐stimulated gene (ISG) expression, T‐cell activation thresholds, or serum cytokine profiles (e.g., interferon‐gamma [IFN‐γ], interleukin‐6 [IL‐6])—in nonclinical studies can provide critical insights for predicting sex‐biased efficacy or immune‐related adverse events in the clinic.^29^, ^45^

In metabolic and endocrine systems, omics analyses across skeletal muscle, liver, and adipose tissue reveal systematic sex differences in glucose/lipid metabolism, mitochondrial pathways, fatty acid oxidation, and insulin signaling.^26^, ^27^ In nonalcoholic fatty liver disease (NAFLD), sex differences in subtype distribution and progression patterns have been linked to endocrine environments and lipidomic profiles.^28^ This implies that nonclinical toxicity assessments for metabolic disorders should include sex‐stratified measures of hepatic lipid accumulation (e.g., triglyceride content, expression of lipogenic genes) and mitochondrial function to identify sex‐dependent susceptibilities to drug‐induced steatosis or insulin resistance.^26^ Cardiovascular omics investigations similarly suggest sex‐dependent pathways influencing disease onset and outcomes.^25^ In cardiotoxicity screening, sex‐aware evaluation of electrophysiological parameters (e.g., action potential duration in cardiomyocytes), cardiac hypertrophy markers, or sex‐specific circulating biomarkers (e.g., NT‐proBNP) can enhance the detection of sex‐biased adverse effects that may be missed in pooled analyses.^19^, ^25^

In kidney research, epidemiological sex differences in chronic kidney disease (CKD) are well recognized, and multiple frameworks propose incorporating sex information into engineered kidney models and in vitro systems.^34^, ^35^ Explicit consideration of sex‐dependent transporter/enzyme expression and donor sex/hormonal conditions can improve predictive performance for nephrotoxicity and drug transport.^40^ Thus, nonclinical renal safety studies should measure sex‐specific biomarkers of tubular injury (e.g., Kim‐1, clusterin) and glomerular function in addition to standard histopathology, as baseline and drug‐induced expression may differ significantly between sexes.^12^, ^43^

Gut microbiota–host interactions represent an emerging frontier, with sex‐associated differences in bile acids and short‐chain fatty acids contributing to metabolic, cardiovascular, and immune phenotypes.^33^, ^51^ For orally administered drugs or those metabolized by gut microbiota, nonclinical studies could benefit from assessing sex differences in gut permeability, bacterial composition, and levels of microbial metabolites (e.g., secondary bile acids) as potential modifiers of drug bioavailability and local toxicity.^33^

Although the networks described above illustrate fundamental biological differences, their direct impact on drug development and safety assessment must be systematically translated. The following section therefore highlights evidence that directly informs pharmacokinetic variability, toxicity risk, and efficacy outcomes—areas critical for decision‐making in nonclinical and early clinical development.

#### 2.2.2 Evidence of particular importance for drug development and toxicity evaluation

Large‐scale analyses of approved drugs provide compelling evidence that women are more likely to experience increased drug exposure and delayed elimination, correlating with sex‐biased adverse drug reaction (ADR) frequencies.^49^, ^50^ For instance, a systematic review demonstrated that women experience average maximum plasma concentrations (C~max~) approximately 10%–20% higher and longer half‐lives for numerous drugs, a PK disparity statistically linked to their higher ADR incidence.^50^ Underlying mechanisms frequently involve reduced hepatic metabolism (particularly for CYP3A4 substrates) and renal clearance observed in females. For anticancer agents, secondary analyses of clinical studies and registries demonstrate sex differences in both efficacy and toxicity, consistent in part with sex‐dependent PK, immune responses, and DNA repair mechanisms.^46^, ^47^ Meta‐analyses of immune checkpoint inhibitor therapies reveal that female patients have a higher incidence of immune‐related adverse events (irAEs), whereas therapeutic efficacy can vary by sex depending on the cancer type.^45^, ^46^ These divergences may be driven by sex hormones modulating immune cell function and differential expression of X‐linked immune regulatory genes. For cardiovascular and hypertension‐related conditions, sex differences in mechanisms and clinical treatment patterns have been summarized.^42^ For major antihypertensive drug classes, such as ACE inhibitors and calcium channel blockers, variations in blood pressure‐lowering efficacy and side effect profiles are documented between men and women.^42^ Mechanistically, premenopausal women often exhibit lower activity of the renin‐angiotensin system compared to age‐matched men, which may contribute to differential responses to ACE inhibitors.^41^ Furthermore, sex‐dependent renal transporter biology provides an important mechanistic bridge to differential drug handling and safety.^41^, ^42^, ^43^

Recent efforts quantify sex differences in enzyme/transporter expression using RNA sequencing and proteomics and integrate these parameters into PK/PD modeling, enabling sex‐aware prediction and stratified decision‐making. Collectively, these findings underscore that clinically observed sex differences are not merely associative but are rooted in fundamental variations in PK, target expression, and host response networks. Consequently, the systematic evaluation of these sex‐specific factors from the nonclinical stage is critical for de‐risking clinical development and enabling stratified medicine.

#### 2.2.3 Implications for nonclinical study design

These findings collectively demonstrate that sex differences are intrinsic biological properties rather than exceptional phenomena. Table 2 provides a map of conserved sex‐dependent networks across immune, metabolic, cardiovascular, renal, and neural systems, whereas Table 3 illustrates how these networks manifest as differences in drug efficacy and toxicity.

Therefore, nonclinical studies should incorporate sex from the design stage, shifting from post hoc explanations to prediction‐oriented, stratified decision‐making.

## 3 SEX DIFFERENCES FROM THE PERSPECTIVE OF ANIMAL WELFARE AND THE 3RS

Sex bias in nonclinical research affects not only scientific validity and human extrapolation but also the ethical framework of animal experimentation (the 3Rs). A central practical question is how to align regulatory expectations for sex consideration with Replacement, Reduction, and Refinement. First, male‐biased study designs can structurally generate surplus animals of the unused sex in breeding colonies. In many mammals, the sex ratio at birth is approximately balanced; therefore, plans presuming male‐only use can reduce the utilization of female offspring and increase culling.^4^ This concern is relevant to facility‐level animal management and to the principle of Reduction when evaluated at the project or colony level rather than within a single experiment.

Second, research sex bias can slow the accumulation of welfare‐relevant knowledge specific to females. Pain‐related behaviors, nociceptive sensitivity, stress responses, and physiological impacts of housing conditions may differ by sex.^52^, ^53^, ^54^ Without sex‐aware study designs, refinement of husbandry and endpoints may remain incomplete for one sex.

A common objection is that including both sexes will increase group size and total animal use. However, interpreting Reduction solely as minimizing the number of animals in a single experiment risks ignoring optimization across the entire development program. Animal studies are justified by a harm–benefit analysis. If sex‐ignorant designs reduce clinical predictability or miss sex‐biased toxicities, additional confirmatory studies or program failures may occur. These consequences may increase cumulative animal use and harm. In this context, sex‐aware design early in development can reduce downstream repetition, improve interpretability, and ultimately decrease total animal use across the project lifecycle.

Thus, sex‐aware design aligns with the scientific rationale summarized in Section 2 and supports welfare rationality by minimizing retesting and cumulative animal burden. These considerations are now formalized as regulatory expectations and funding requirements, moving beyond discretionary recommendations.

## 4 SEX DIFFERENCES FROM THE PERSPECTIVE OF REGULATORY EXPECTATIONS

Building on this understanding, the following section examines how these expectations are embedded in current regulatory frameworks across major jurisdictions.

### 4.1 International Council for Harmonization guidelines (international)

The International Council for Harmonization (ICH) guidelines provide a shared foundation for nonclinical and clinical development across major regulatory authorities. ICH M3(R2) defines timing and baseline requirements for nonclinical safety studies and states that, in principle, repeated‐dose toxicity studies should include animals of both sexes.^55^ Similarly, for biotechnology‐derived pharmaceuticals, ICH S6(R1) requires safety evaluation in pharmacologically relevant species, typically involving both sexes, to assess potential immunogenicity and off‐target effects that may differ by sex. Single‐sex designs are generally limited to sex‐specific indications (e.g., prostate cancer, endometriosis) or cases where limited sex impact can be scientifically justified.^56^ Thus, sex consideration is positioned as a default prerequisite in nonclinical safety evaluation rather than an optional add‐on. Revisions such as ICH E8(R1) further emphasize representativeness and generalizability in clinical development, reinforcing the expectation that sex‐related logic should be continuous from nonclinical evidence to clinical trial design.^57^

### 4.2 United States (NIH/FDA)

In the United States, sex consideration is institutionalized through both NIH funding requirements and regulatory review expectations. The NIH introduced its SABV policy in 2016, requiring sex to be addressed as a biological variable in vertebrate animal research.^5^ In parallel, the Food and Drug Administration (FDA), through its Office of Women's Health, actively promotes the evaluation of sex differences in research and development. Although the FDA increasingly promotes New Approach Methodologies (NAMs) and non‐animal approaches, representativeness and generalizability remain central.^58^ Sex‐biased data can fail to represent diverse patient populations, even when derived from non‐animal systems, thereby reinforcing the importance of sex‐aware evidence structures.

### 4.3 Europe (European Medicines Agency (EMA))

European frameworks, grounded in Directive 2010/63/EU, emphasize that the 3Rs and scientific validity are complementary, not opposing. The Directive explicitly requires that animal experiments be designed to yield the most satisfactory results with the minimum number of animals, implying that excluding one sex without justification violates the principle of optimal design. Poorly designed studies aimed only at reducing animal numbers can decrease reproducibility, necessitate additional studies, and ultimately increase animal use. In this context, the SAGER guidelines are widely supported as a reporting framework to improve transparency and translational value.^7^

### 4.4 Japan (Pharmaceuticals and Medical Devices Agency (PMDA))

In Japan, regulatory practice aligns with international trends. Under ICH‐based reviews, the completeness and interpretability of sex‐disaggregated nonclinical data become critical when sex differences in PK or safety are anticipated. National development policy discussions similarly emphasize that ignoring sex differences at early stages may increase attrition risk in later development, particularly in disease areas with marked sex differences.

### 4.5 China (National Medical Products Administration (NMPA))

As an ICH member, China's regulatory environment prioritizes international harmonization. Although sex‐specific requirements may not be uniquely detailed compared with other jurisdictions, the expectation for scientific justification and transparent evidence structure is increasing in step with global development strategies.^59^

### 4.6 Sex consideration in the NAMs era: In silico and organ‐on‐chip

The expansion of NAMs—such as in silico modeling, human cell–based in vitro systems, and organ‐on‐chip technologies—supports Replacement and Reduction.^60^

At the same time, it introduces new design requirements for biological representativeness. In silico models depend on input parameters (e.g., enzyme activity, transporter abundance, endocrine conditions) that must be explicitly linked to the represented population, including sex. Similarly, organ‐on‐chip and human cell systems require explicit reporting and consideration of donor sex, hormonal conditions, and culture parameters.

A critical barrier in current in vitro modeling is the historical bias in cell line availability; for instance, many widely used immortalized cell lines are derived from male donors, and there is a documented scarcity of well‐characterized female‐derived lines for certain organ systems. This can lead to sex‐biased results in toxicological screening. Consequently, a single‐donor, single‐sex model can reproduce the same representativeness limitations as a single‐sex animal design.

To address these gaps, physiologically based pharmacokinetic (PBPK) modeling platforms, such as GastroPlus and Simcyp Animal, now allow for the integration of sex‐specific physiological parameters (e.g., organ volumes, blood flow) to simulate and predict sex‐dependent drug distribution and clearance.^61^, ^62^ By leveraging these computational tools alongside sex‐aware in vitro systems, researchers can better extrapolate nonclinical findings to a diverse human population.

### 4.7 Case studies: Lessons from sex‐aware and sex‐ignorant nonclinical programs

The following case studies present illustrative scenarios informed by published evidence and regulatory experience, highlighting the potential consequences of integrating—or overlooking—sex as a biological variable in nonclinical drug development. These scenarios are synthesized for illustrative purposes and are not intended to describe any single specific development program.

#### 4.7.1 Case study 1: Immune checkpoint inhibitor—Averted toxicity through early sex inclusion

This is a hypothetical scenario synthesized for illustrative purposes and does not describe any single specific development program.

During the development of a novel PD‐1 inhibitor, initial exploratory toxicity studies were conducted in male mice only, revealing no significant hepatotoxicity. However, when the study was repeated including both sexes under an SABV‐compliant protocol, female mice exhibited a significant incidence of immune‐mediated hepatitis, characterized by elevated transaminases and histopathological findings.^63^ Mechanistic follow‐up was consistent with female‐biased activation of interferon‐gamma pathways in hepatic lymphocytes.^29^ This nonclinical observation supported enhanced liver function monitoring in early clinical studies, which may facilitate earlier detection and management of similar events in female patients. This case underscores how sex‐aware design can uncover clinically relevant toxicities that single‐sex studies may miss.

#### 4.7.2 Case study 2: Analgesic drug—Pharmacokinetic sex difference informing clinical dosing

This is a hypothetical scenario synthesized for illustrative purposes and does not describe any single specific development program.

A candidate analgesic targeting the central nervous system showed promising efficacy in male rats. However, subsequent PK studies in both sexes indicated that female rats had substantially lower systemic clearance, leading to higher and more sustained drug exposure. This difference was consistent with lower expression of a key CYP3A isoform in female rat livers.^11^ A PBPK model incorporating these sex‐specific parameters suggested that a comparable exposure difference could occur in humans.^64^ As a result, the clinical trial protocol was designed with sex‐stratified PK analysis and a lower starting dose for female participants, potentially reducing the risk of dose‐related sedative adverse events. This example demonstrates how early PK sex comparison can directly inform safer clinical trial design.

#### 4.7.3 Case study 3: Neurodegenerative disease model—The risk of missed efficacy

This is a hypothetical scenario synthesized for illustrative purposes and does not describe any single specific development program.

In a transgenic mouse model of Alzheimer's disease, a therapeutic antibody significantly reduced amyloid plaque load in males, supporting progression into later development stages. However, when efficacy was later evaluated in females as part of a regulatory request, the treatment effect was markedly attenuated. Post‐hoc analyses were consistent with female‐specific neuroinflammatory responses that could mitigate the drug's plaque‐clearing mechanism.^65^ This delay in sex‐aware testing not only consumed additional resources but also raised questions about the program's viability. It highlights that failing to evaluate both sexes early can lead to an overestimation of efficacy and late‐stage attrition.

These case studies collectively illustrate that the upfront investment in sex‐aware nonclinical studies is often offset by mitigating downstream risks, including clinical trial failures, post‐marketing safety issues, and redundant animal testing.

## 5 PRACTICAL CHECKLIST FOR IMPLEMENTING SABV IN NONCLINICAL STUDY DESIGN

**FIGURE 2:**
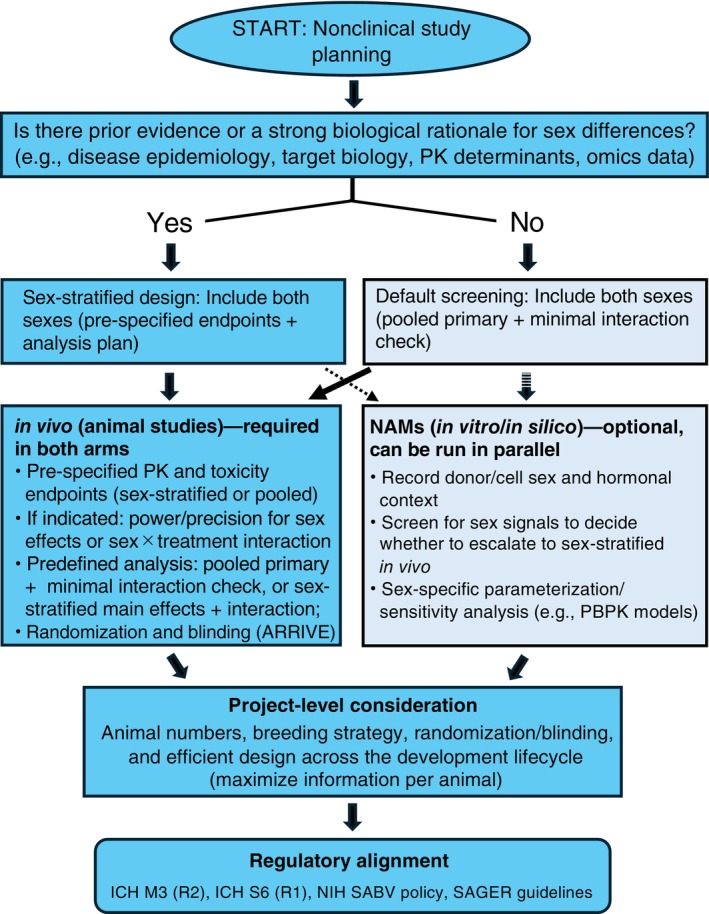
Decision‐making flowchart for implementing sex as a biological variable (SABV) in nonclinical research. This schematic summarizes a pragmatic, evidence‐anchored approach to sex‐aware nonclinical study design. If prior evidence or strong biological rationale suggests sex differences (e.g., epidemiology, target biology, pharmacokinetic determinants, omics), studies should use a sex‐stratified design including both sexes, with prespecified endpoints and an analysis plan to test sex effects and, where relevant, sex × treatment interactions. If such evidence is lacking, a screening approach can still include both sexes while prioritizing efficiency, typically using pooled primary analyses with a minimal interaction check. In parallel, NAMs (New Approach Methodologies; in vitro/in silico) can help detect sex signals and guide whether escalation to fully sex‐stratified in vivo studies is warranted. NAMs include a range of non‐animal approaches such as in silico models, organ‐on‐chip technologies, and human cell‐based in vitro assays. Practical considerations—animal numbers, breeding strategy, randomization and blinding (e.g., ARRIVE), and maximizing information gain per animal—support robust, reproducible, welfare‐conscious decisions. Solid arrows indicate the required decision path; dotted arrows indicate optional/parallel NAM use in either arm.

A schematic overview of this practical checklist is shown in Figure 2.

1. *Hypothesis*: Clarify the sex‐difference hypothesis based on indication, target biology, pathway, and existing epidemiologic/omics evidence.
2. *Design Justification*: Define the default use of both sexes. Explicitly justify any single‐sex design (e.g., sex‐specific indication, mechanistically limited sex impact).
3. *Stratified Analysis Plan*: Plan sex‐stratified PK and toxicity analyses from early stages. Predefine interpretation rules for observed sex differences.
4. *NAM Specifications*: Specify sex assumptions in NAMs (in silico/in vitro/organ‐on‐chip), including donor sex, endocrine context, and parameterization.
5. *Continuity to Clinical*: Build a continuous sex‐based logic from nonclinical to clinical stages, consistent with ICH E8(R1), linking nonclinical findings to clinical enrollment and stratified analyses.

## 6 FUTURE DIRECTIONS: INTEGRATION OF ARTIFICIAL INTELLIGENCE, MACHINE LEARNING, AND PERSONALIZED PREDICTION

Sex differences are not reducible to hormone levels alone; omics evidence reveals system‐wide molecular network differences spanning immune, metabolic, cardiovascular, detoxification, and other systems. Nonclinical designs that ignore these networks can compromise PK and toxicity reproducibility, increase late‐stage attrition, and necessitate additional studies—thereby increasing cumulative animal use and burden.

Sex‐aware designs are therefore scientifically rigorous and welfare‐rational, supporting both interpretability and 3Rs optimization at the development‐program level. These expectations are increasingly institutionalized through ICH‐based regulatory harmonization and funding requirements, and they remain critical even in the NAMs era, where model representativeness by sex is a central design principle.

The future of SABV implementation lies in the sophisticated integration of big data and computational modeling.

Artificial intelligence (AI) and machine learning (ML) are poised to transform sex‐inclusive research by identifying complex, nonlinear patterns from large omics datasets that are intractable to conventional analysis.

For instance, deep learning models can integrate transcriptomic, proteomic, and metabolomic data from both sexes to predict sex‐specific toxicity signatures for new chemical entities, potentially prioritizing compounds with balanced efficacy and safety profiles across sexes early in discovery.

The next generation of PBPK models will move beyond static sex‐specific parameters to dynamic, agent‐based simulations that incorporate individual variability, including hormonal cycles and genetic polymorphisms. These next‐generation models will enable the creation of “virtual twin” populations that mirror real‐world demographic distributions, improving the accuracy of clinical trial simulations. For example, they can simulate the impact of menstrual or estrous cycles on drug exposure, or account for the population prevalence of sex‐differentially expressed enzymes (e.g., CYP2C19) and transporters. This capability allows for more accurate clinical trial simulations, identification of high‐risk subgroups (e.g., premenopausal women on specific concomitant medications), and optimization of dosing regimens before first‐in‐human studies.^66^

Furthermore, the emergence of sex‐annotated biobanks for cell lines and organoid development will be crucial. Initiatives to derive and characterize induced pluripotent stem cell (iPSC) lines from balanced donor cohorts will help overcome the historical male bias in in vitro models, providing a foundational resource for truly representative NAMs.

From a regulatory perspective, we anticipate evolving guidelines that will not only require the inclusion of both sexes but also the application of computational tools to justify bridging strategies when one sex is underrepresented in a study. The ultimate goal is a paradigm shift toward predictive stratified toxicology, where nonclinical testing is designed from the outset to inform personalized medicine, ensuring drug safety and efficacy for all patient subpopulations.

Accordingly, sex consideration should be positioned not as an additional burden but as a core design requirement and a catalyst for innovation. It simultaneously optimizes nonclinical quality, advances animal welfare, ensures regulatory fitness, and paves the way for a more predictive and equitable drug development ecosystem.

## AUTHOR CONTRIBUTIONS

**Kenta Onuma:** Investigation; visualization; writing – original draft. **Masaki Watanabe:** Investigation; validation; writing – original draft. **Nobuya Sasaki:** Investigation; supervision; visualization; writing – original draft; writing – review and editing.

## FUNDING INFORMATION

This research received no external funding.

## CONFLICT OF INTEREST STATEMENT

The authors declare no conflicts of interest.

## ETHICS STATEMENT

Not applicable. This article is a review and does not contain any new studies involving human participants or animals performed by the authors.

## Data Availability

None.
